# Rifaximin Plus Probiotics Reshape Gut Microbiota, Serum Propionate, and Mucosal Immunity in Cirrhosis‐Related Hepatic Encephalopathy Microbiota‐Immune Remodeling in HE

**DOI:** 10.1155/cjgh/2389961

**Published:** 2026-07-13

**Authors:** Yiping Wang, Hui Zhu, Shujun Wang, Danni Hu, Mengdan Xu, Luna Lee, Dylan Lee

**Affiliations:** ^1^ Department of Gastroenterology, Affiliated Cixi Hospital, Wenzhou Medical University, Cixi, Zhejiang, 315300, China, wmu.edu.cn; ^2^ Family Medicine, Axess Family Services, Inc., Ravenna, Ohio, 44226, USA; ^3^ Internal Medicine, CuraStrategix Health, Cleveland, Ohio, 44202, USA

**Keywords:** hepatic encephalopathy, microbiota, secretory IgA, short-chain fatty acid

## Abstract

**Background:**

Hepatic encephalopathy (HE) remains a major cause of hospitalization and readmission in cirrhosis and is closely linked to hyperammonemia, microbial dysbiosis, impaired short‐chain fatty acid (SCFA) output, barrier dysfunction, and altered mucosal immunity. We evaluated whether adding a multistrain probiotic to rifaximin was associated with greater neurometabolic improvement and coordinated gut–liver–brain axis changes.

**Methods:**

In this prospective, 6‐month, randomized, open‐label, assessor‐blinded, three‐arm controlled study, 61 adults with cirrhosis‐related HE received standard care alone (Con, *n* = 20), standard care plus rifaximin 550 mg twice daily (Rif, *n* = 20), or rifaximin plus a multistrain probiotic (1 × 10^9 CFU three times daily; Rif + Pro, *n* = 21). The main prespecified biochemical readout was serum ammonia at Month 6. A composite HE index incorporating mental status, flapping tremor, number connection test, and ammonia grade was analyzed as a prespecified exploratory neurometabolic score. A predefined mechanistic subset underwent 16S rRNA microbiome profiling, serum SCFA measurement, and fecal secretory IgA (SIgA) testing.

**Results:**

Baseline characteristics did not differ across arms. Post‐treatment serum ammonia decreased in a graded pattern (Con 177 ± 43.2, Rif 143 ± 37.5, Rif + Pro 117 ± 34.3 μmol/L), with parallel improvement in the exploratory HE index (10.0 ± 4.9, 5.3 ± 4.7, and 3.7 ± 4.1, respectively). In the mechanistic subset, the microbial community structure differed by treatment (PERMANOVA *p* = 0.006). Rif + Pro was associated with higher serum propionate, increased Lactobacillus salivarius–associated signal, reduced Bacteroides ovatus–associated signal, and marked fecal SIgA elevation compared with standard care or rifaximin alone. The SIgA–propionate relationship was interpreted as exploratory.

**Conclusions:**

Rifaximin plus multistrain probiotics was associated with greater improvement in serum ammonia and exploratory HE severity readouts than rifaximin alone, accompanied by coordinated microbial, metabolic, and mucosal immune changes. These findings support confirmation in adequately powered event‐driven trials with strain‐resolved profiling and targeted metabolomics.

**Highlights**
 In cirrhosis‐related hepatic encephalopathy, rifaximin plus multistrain probiotics showed the strongest serum ammonia reduction and exploratory HE‐index improvement, with distinct microbiota states, higher serum propionate, and markedly increased fecal SIgA versus standard care or rifaximin alone. These findings support event‐driven confirmation rather than definitive efficacy claims.

## 1. Introduction

Hepatic encephalopathy (HE) is a major neuropsychiatric complication of cirrhosis and portosystemic shunting, manifesting as cognitive slowing, behavioral and sleep–wake disruption, and neuromotor dysfunction that can progress to coma [1–3]. Beyond its immediate morbidity, HE is a common driver of hospitalization, early readmission, and sustained caregiver and healthcare‐system burden in cirrhosis [4]. Current standard therapy, lactulose for secondary prophylaxis and rifaximin for recurrent episodes, reduces recurrence, yet many patients continue to experience residual cognitive impairment and recurrent decompensation. These persistent deficits emphasize that HE is not only an episodic intoxication syndrome but also a system‐level disorder of the gut–liver–brain axis that may require interventions capable of reshaping upstream biology rather than only treating downstream symptoms.

Hyperammonemia remains a central pathophysiologic contributor, but ammonia alone does not fully explain the heterogeneity of clinical severity or the persistence of neurocognitive dysfunction after apparent clinical stabilization [2]. In cirrhosis, impaired hepatic detoxification coincides with increased extrahepatic ammonia generation, in part mediated by microbial urease and nitrogen metabolism, while barrier dysfunction and altered intestinal permeability permit microbial products and metabolites to enter the portal and systemic circulation [1, 5]. Ammonia can cross the blood–brain barrier and is converted to glutamine within astrocytes, promoting osmotic stress, oxidative injury, neurotransmission imbalance, and neuroinflammatory signaling that culminate in cerebral metabolic dysfunction [6]. This integrated model motivates therapeutic strategies that pair ammonia‐lowering with interventions that stabilize the intestinal ecosystem, reinforce barrier integrity, and attenuate inflammatory signaling along the gut–liver–brain axis.

Cirrhosis and HE are consistently associated with gut microbiota perturbations, including depletion of fermentative commensals and enrichment of taxa linked to inflammation, endotoxin exposure, and nitrogenous metabolism, alongside functional loss of short‐chain fatty acid (SCFA) production capacity [3]. SCFAs, particularly acetate, propionate, and butyrate, are key microbial fermentation outputs that influence epithelial energy metabolism, tight‐junction integrity, immune calibration, and hepatic metabolic homeostasis [7–14]. SCFAs also signal systemically and can influence neuroimmune pathways, including microglial state and neuroinflammatory tone, thereby providing a plausible mechanistic bridge between intestinal dysbiosis and cerebral vulnerability in HE [15, 16]. In this context, the clinical relevance of microbiome interventions may depend less on restoring “diversity” per se and more on reconfiguring ecosystem function toward fermentative outputs and away from urease‐ and endotoxin‐associated metabolic programs.

Mucosal immunity represents a complementary and under‐measured axis of this biology. Secretory immunoglobulin A (SIgA), the dominant immunoglobulin at mucosal surfaces, coats microbes and antigens, limits epithelial adhesion and penetration, and shapes microbial community structure while dampening inflammatory exposure [12, 17–20]. In cirrhosis, immune dysfunction is characterized by the coexistence of impaired surveillance and maladaptive activation, and disruption of SIgA quantity or function can destabilize microbial networks and facilitate translocation of microbial products [17, 19]. Several probiotic strains and consortia have been reported to augment mucosal IgA responses in humans, although effects are strain‐specific and require careful safety attention in cirrhosis, particularly when formulations include organisms with theoretical bacteremia risk [16, 21]. Critically, few HE studies have evaluated SIgA as a mechanistic readout in parallel with microbial composition and metabolic outputs, limiting our ability to link microbiota‐targeted therapies to mucosal immune restoration in vivo.

Rifaximin provides a pragmatic anchor therapy in this space. As a poorly absorbed antibiotic, it reduces HE recurrence and is thought to modulate microbial function and toxin production, yet available evidence suggests that it does not reliably restore beneficial fermentative networks or microbial metabolite profiles across patients [1, 3]. This creates a biologically grounded rationale for combination strategies: rifaximin may suppress dysbiotic nitrogen flux and proinflammatory microbial activity, while a multistrain probiotic could help re‐establish fermentative cross‐feeding, promote SCFA output, and support SIgA‐mediated mucosal immune surveillance. However, despite widespread clinical interest in microbiota‐targeted approaches (prebiotics, probiotics, fecal microbiota transplantation) [1, 3], there remains a translational gap: human studies rarely integrate, within the same cohort and time window, (i) clinical neurometabolic readouts, (ii) community‐level ecology, (iii) circulating microbial metabolites such as SCFAs, and (iv) mucosal immune measures such as SIgA, particularly under a comparative design that can distinguish rifaximin monotherapy from combination therapy.

To address this gap, we conducted a prospective, randomized, open‐label, assessor‐blinded, three‐arm, 6‐month study comparing routine management alone, rifaximin, and rifaximin plus a multistrain probiotic in adults with cirrhosis‐related HE. We evaluated serum ammonia as the main prespecified biochemical readout and a composite HE index incorporating mental status, asterixis, number connection testing, and ammonia grading as a prespecified exploratory neurometabolic score. Prespecified mechanistic measures in a predefined subset included fecal microbiota community structure, serum SCFA profiles, and fecal SIgA. By aligning neurometabolic gradients with ecological, metabolic, and mucosal immune signals under a comparative intervention framework, this study tested whether combination microbiota‐targeted therapy was associated with additive biological improvement beyond rifaximin alone and characterized coordinated microbial–metabolic–immune changes across the gut–liver–brain axis.

## 2. Methods

### 2.1. Study Design and Ethics

We conducted a prospective, randomized, open‐label, assessor‐blinded, three‐arm controlled study between December 2018 and November 2022. Adults with cirrhosis and a history of HE were screened after clinical evaluation and stabilization of immediately reversible precipitants when present. Cirrhosis was diagnosed based on clinical history and radiologic/clinical findings. HE severity was graded using Conn’s modification of the Parsons–Smith/West Haven classification. Patients were eligible if they were clinically stable for study participation at randomization, including those with West Haven/Conn mental‐state grades 1–3. Patients with grade 4 HE/coma, uncontrolled acute precipitating factors, active infection, ongoing gastrointestinal bleeding, severe or unstable renal impairment, known intracranial structural lesions, inability to complete neurologic assessment, inability to provide informed consent, or conditions limiting adherence were excluded. Probiotic or antibiotic therapy targeting HE within 8 days prior to enrollment was not permitted, and the interval from the most recent overt HE episode was recorded. Baseline HE grade in Table 1 refers to mental‐state grade assessed at randomization after screening and stabilization procedures. Ethical approval was obtained from the institutional Ethics Review Committee (No. 2018‐LP‐19). All participants provided written informed consent in accordance with the Declaration of Helsinki. Reporting followed applicable observational/interventional reporting guidance where relevant [22].

**TABLE 1:** Baseline HE characteristics of patients.

	Con (*n* = 20)	Rif (*n* = 20)	Rif + Pro (*n* = 21)	*p* value
Baseline mental state grade (0/1/2/3/4)	0/12/5/3/0	0/13/3/4/0	0/13/4/4/0	0.504
Serum ammonia (μmol/L)	200.4 ± 47.1	197.8 ± 62.1	212 ± 54.7	0.657

*Note:* Serum ammonia concentration was presented as mean ± SD.

Abbreviations: Con, routine treatment; Rif, routine treatment + rifaximin; Rif + Pro, routine treatment + rifaximin + probiotics.

### 2.2. Participants and Interventions

Participants were randomized in a 1:1:1 ratio to (i) standard of care (Con), (ii) standard‐of‐care plus rifaximin (Rif), or (iii) standard‐of‐care plus rifaximin and a multistrain probiotic (Rif + Pro). Standard of care included lactulose titrated to two to three soft stools/day, diuretics as clinically indicated, nonselective *β*‐blockers when appropriate, endoscopic evaluation per portal‐hypertension practice, and dietary sodium restriction.

Randomization used a computer‐generated permuted‐block sequence with variable block sizes, prepared by an independent statistician. Allocation was implemented using sequentially numbered, sealed, opaque envelopes. Outcome assessors performing neurologic testing and investigators conducting data analysis were blinded to group assignment.

Rifaximin was administered at 550 mg twice daily. The probiotic was administered at 1 × 10^9 CFU three times daily for 6 months. The formulation contained Lactobacillus plantarum, L. acidophilus, L. paracasei, Leuconostoc mesenteroides, L. bulgaricus, L. casei, L. salivarius, Pediococcus pentosaceus, *Streptococcus* thermophilus, *Bacillus* subtilis, B. coagulans, *Enterococcus* faecium, Bifidobacterium bifidum, B. longum, B. infantis, and B. animalis subsp. lactis. Certificates of Analysis for identity and viable counts and manufacturing quality‐control documentation are provided in the Supporting information. Given cirrhosis‐related infection risk and the inclusion of E. faecium, safety surveillance was prespecified: weekly symptom checks during Month 1, monthly laboratory monitoring thereafter (CBC and comprehensive metabolic panel), and blood cultures if fever or systemic infection was suspected. No prophylactic antibiotics were used beyond rifaximin in the allocated arms.

### 2.3. Outcomes and Follow‐Up Schedule

The main prespecified biochemical readout was post‐treatment serum ammonia concentration at Month 6, analyzed as a continuous variable. The composite HE index (3 × mental state grade + flapping tremor grade + NCT grade + ammonia grade) was prespecified as an exploratory neurometabolic severity score to summarize HE‐related neurologic, psychometric, and biochemical domains for internal comparison. It was not interpreted as a validated hard clinical endpoint for recurrence, hospitalization, transplant, or survival. Mechanistic readouts were fecal microbiota composition (16S rRNA sequencing), fecal secretory IgA (SIgA), and serum SCFAs (including acetate, propionate, butyrate, and branched‐chain SCFAs). Study visits occurred at baseline and Months 1, 3, and 6. Baseline and Month‐6 values supported the principal comparative analyses, whereas Month 1 and Month 3 contacts captured adherence, tolerability, suspected infection, adverse events, hospitalization, and treatment discontinuation for safety and feasibility description. Health‐related quality‐of‐life outcomes were not included in the final analytic dataset and are therefore not reported as study endpoints. HE‐related rehospitalization and death were recorded when they occurred during surveillance but were not powered or analyzed as formal time‐to‐event efficacy endpoints in the present mechanistic study.

Serum ammonia was analyzed as a continuous variable, with descriptive categories (≤ 60, 61–100, > 100 μmol/L) used for visualization only. The composite HE index was reported strictly as an exploratory neurometabolic readout. The mechanistic subset was predefined based on the availability of paired high‐quality stool and serum specimens collected within the required processing window. The subset included 20 participants: Con *n* = 7, Rif *n* = 7, and Rif + Pro *n* = 6. Because of the limited subset size, microbiome, SCFA, and SIgA analyses were considered exploratory and hypothesis‐generating.

### 2.4. Clinical and Neurologic Assessments

Clinical and neurologic assessments were performed by an experienced gastroenterologist and independently reviewed by a second senior gastroenterologist; discrepancies were resolved by consensus and documented. Mental status was graded using Conn’s modification of the West Haven criteria. Asterixis (flapping tremor) was graded per Conn. Psychometric performance was assessed with NCT‐A under standardized instructions. Child–Pugh class/score and MELD‐Na were recorded at baseline along with hemoglobin, white cell count, platelets, INR, total bilirubin, sodium, albumin, creatinine/eGFR, CRP, and prior OHE history. Lactulose dose and adherence were documented at each visit [1, 3, 4].

### 2.5. Sample Collection and Processing

Fasting venous blood was collected on ice; serum was aliquoted within 30 min and stored at −80°C until analysis. Stool was collected in sterile containers, frozen within 2 h of passage, and stored at −80°C. Serum was used for SCFA and ammonia assays, and stool was used for microbiome profiling and fecal SIgA measurement. Unless otherwise specified, laboratory measurements were performed in triplicate per sample, with plate‐level quality control and predefined acceptance ranges.

### 2.6. Fecal SIgA Measurement

Stool samples were thawed on ice and processed in batch. Approximately 100 mg of stool was homogenized in extraction buffer, centrifuged (10,000 × g, 10 min), and supernatants were diluted 1:250 in assay buffer. SIgA was quantified by ELISA using a human secretory IgA kit (Biovendor, Cat. No. RIC6100R, Guangzhou, China) following the manufacturer’s protocol. Each plate included kit standards and pooled‐stool internal controls; all samples were measured in triplicate.

### 2.7. SCFA Quantification and Ammonia Assay (Serum)

Serum SCFAs were quantified by UPLC‐ESI‐MS/MS using a Nexera UHPLC LC‐30A system (Shimadzu, Kyoto, Japan) coupled to a QTRAP 5500 mass spectrometer (SCIEX/AB Sciex, Framingham, MA, USA). Analyses were performed by an accredited laboratory (Shanghai Luming Biological Technology Co., Ltd., Shanghai, China) under validated standard operating procedures covering protein removal, derivatization, chromatographic separation, instrument calibration, and quality control; SOP summaries are available upon request. Serum ammonia was measured using the Ammonium Detection Kit (Saint‐Bio, Cat# BA1887, Shanghai, China) after protein precipitation and conversion to ammonium salts, with absorbance read at 630 nm. Reagent blanks, calibration curves, and internal standards were included in each run; analytes were assayed in triplicate.

### 2.8. 16S rRNA Sequencing, Bioinformatic Processing, and Community Ecology

DNA was extracted using QIAamp DNA Stool Mini Kit (QIAGEN, Cat. 51504) and/or DNeasy PowerSoil Kit (QIAGEN, Cat. 12888–50/12888–100) according to the manufacturer’s protocols; AMPure XP beads (Beckman Coulter, Cat. A63880), Qubit dsDNA HS (Thermo Fisher, Cat. Q32851), and NanoDrop 2000 (Thermo Fisher, Cat. ND‐2000) were used for cleanup and quantification as appropriate.

Raw FASTQ reads were adapter‐trimmed with cutadapt, quality‐filtered, denoised, merged, and chimera‐checked using DADA2 within QIIME 2 (v2020.11), generating amplicon sequence variants (ASVs) and an ASV abundance table [23]. Taxonomy was assigned against SILVA v138 using the QIIME 2 feature‐classifier plugin, and a phylogenetic tree was constructed within QIIME 2. Alpha diversity was quantified using Simpson, Shannon, and Chao1 indices. Beta diversity was assessed using unweighted UniFrac distances, with ordination by PCoA; NMDS ordination was additionally performed as a complementary low‐dimensional representation where indicated.

To support ecological visualization, rank‐abundance curves were constructed from within‐sample relative abundance ranked by ASV/OTU abundance, and species/OTU accumulation curves were generated to summarize feature discovery as a function of the sample number. Ternary plots were used to visualize compositional bias across treatment arms at the specified taxonomic level. For discriminant feature discovery, LEfSe was used to identify differentially enriched taxa with effect sizes (LDA scores), and indicator value analysis was performed to identify ASVs with group‐specificity (methods and thresholds specified in Supporting Methods if required by the journal). A random forest classifier was used for feature importance ranking (MeanDecreaseGini) to prioritize genera contributing to group discrimination.

### 2.9. Statistical Analysis

Analyses were performed in R. Continuous variables are reported as mean ± SD, and categorical variables are reported as counts and percentages. Between‐group comparisons for the main biochemical readout and exploratory HE index used one‐way ANOVA or Kruskal–Wallis tests with Bonferroni‐adjusted pairwise comparisons as appropriate; categorical outcomes used *χ* ^2^ or Fisher’s exact tests. Within‐group pre‐ versus post‐treatment comparisons used paired *t*‐tests or Wilcoxon signed‐rank tests. Microbial beta‐diversity differences across groups were tested by PERMANOVA (999 permutations), and multiple comparisons were controlled using the Benjamini–Hochberg false discovery rate for post hoc tests when applicable. Spearman correlation was used for exploratory association analyses between serum propionate and fecal SIgA. All reported *p* values are two‐sided. Two‐sided *p* < 0.05 was considered statistically significant unless otherwise specified. Mechanistic subset analyses (microbiome, SCFAs, SIgA) were prespecified as exploratory, with FDR control where applicable.

No formal time‐to‐event efficacy analysis was performed because HE‐related rehospitalization or death was not a powered formal efficacy endpoint in this mechanistic study.

## 3. Results

Study population, randomization, and follow‐up completion

Between December 2018 and November 2022, 61 clinically stabilized adults with cirrhosis and a history of HE were enrolled and randomized in a 1:1:1 ratio to routine standard‐of‐care (Con, *n* = 20), standard‐of‐care plus rifaximin (Rif, *n* = 20), or standard‐of‐care plus rifaximin combined with a multistrain probiotic (Rif + Pro, *n* = 21). All participants completed the planned 6‐month observation without withdrawal or major protocol deviation, and adherence to background lactulose and allocated interventions was monitored at each visit. No nonprotocol, rifaximin‐unrelated systemic antibiotic exposure was recorded during follow‐up, reducing the likelihood that mechanistic readouts were driven by external antibiotic confounding rather than assigned therapy.

Baseline comparability and analytic integrity

Baseline demographics, cirrhosis etiology, liver disease severity (Child–Pugh score/class), mental‐state grade at randomization, and baseline serum ammonia did not differ across arms (Tables 1 and 2). Baseline mental‐state grades in Table 1 were assessed after screening and stabilization procedures. Therefore, grade 2–3 participants listed in Table 1 represent clinically stable participants with persistent HE manifestations at enrollment, not patients with uncontrolled acute HE requiring urgent inpatient management. Data completeness was sufficient for the analyses reported in Tables 1, 2, 3, and no clinically meaningful baseline imbalance requiring post hoc covariate correction was identified.

**TABLE 2:** Demographic data and baseline cirrhosis characteristics of patients.

	Con (*n* = 20)	Rif (*n* = 20)	Rif + Pro (*n* = 21)	*p* value
Age (years)	44 ± 7.5	40 ± 10.8	42 ± 7.8	0.773
Gender (M/F)	13/7	11/9	12/9	0.897
Cause of liver disease (viral/alcohol/other)	14/3/3	11/5/4	14/4/3	0.671
Child–Pugh score	8.0 ± 1.6	8.3 ± 1.5	8.1 ± 1.6	0.701
Child–Pugh classification (A/B/C)	5/11/4	4/12/4	5/13/3	0.848

*Note:* Values were presented as mean ± SD.

Abbreviations: Con, routine treatment; Rif, routine treatment + rifaximin; Rif + Pro, routine treatment + rifaximin + probiotics.

**TABLE 3:** HE index after treatment.

	Con (*n* = 20)	Rif (*n* = 20)	Rif + Pro (*n* = 21)
Serum ammonia (μmol/L)	177 ± 43.2	143 ± 37.5^∗^	117 ± 34.3^∗∗^
Serum ammonia grade	1.8 ± 0.5	1.1 ± 0.6^∗^	0.8 ± 0.4^∗∗^
Mental state grade	1.2 ± 0.6	0.5 ± 0.3^∗^	0.3 ± 0.3^∗∗^
Flapping tremor test grade	1.4 ± 0.8	0.4 ± 0.7^∗^	0.3 ± 0.7^∗^
NCT test grade	2.7 ± 1.0	2.0 ± 0.8^∗^	1.4 ± 0.9^∗∗^
Total HE index score	10.0 ± 4.9	5.3 ± 4.7^∗^	3.7 ± 4.1^∗∗^

*Note:* Total HE index = 3× (mental state grade) +1× (serum ammonia grade) +1× (flapping tremor test grade) +1× (NCT test grade). The 3× weighting for mental status reflects the central clinical importance of cognitive impairment in hepatic encephalopathy and is intended for descriptive and exploratory use.

^∗^The difference was statistically significant compared to the Con group.

^∗∗^The difference was statistically significant compared to the Rif group.

Neurometabolic response shows a stepwise gradient favoring combination therapy.

Post‐treatment serum ammonia exhibited a graded reduction across arms, consistent with an intervention‐intensity gradient. Mean (±SD) post‐treatment ammonia was highest in Con (177 ± 43.2 μmol/L), intermediate in Rif (143 ± 37.5 μmol/L), and lowest in Rif + Pro (117 ± 34.3 μmol/L), with statistically significant differences for Rif vs Con and for Rif + Pro vs both Con and Rif (*p* < 0.05 for each comparison; Table 3). The exploratory composite HE index mirrored the same monotonic pattern: Con 10.0 ± 4.9, Rif 5.3 ± 4.7, Rif + Pro 3.7 ± 4.1, with significant stepwise contrasts (Con vs Rif and Rif vs Rif + Pro, *p* < 0.05; Table 3). These two measures are presented as supportive neurometabolic readouts rather than definitive clinical endpoints; their internal coherence provides an anchor against which the microbial, metabolic, and mucosal immune readouts can be interpreted.

Mechanistic subset: ecological coverage and community structure indicate treatment‐associated reconfiguration.

**FIGURE 1:**
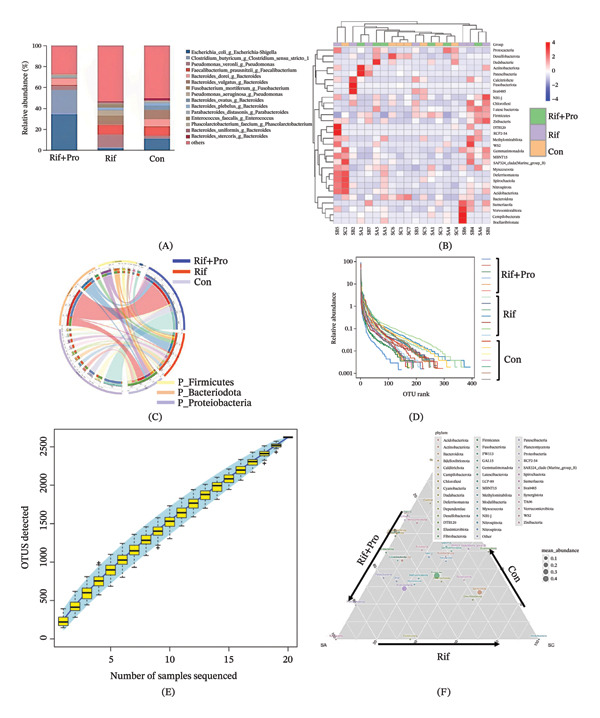
Post‐treatment fecal microbiota landscape, ecological coverage, and compositional bias across treatment arms in the mechanistic subset. (A) Stacked relative abundance of the dominant taxa with “others” pooled, shown by arm (Rif + Pro, Rif, Con). (B) Unsupervised hierarchical clustering heatmap of major lineages (phylum/major groups), with sample annotation by arm; color scale indicates standardized relative abundance. (C) Circos visualization summarizing multiphylum contributions across arms, highlighting prominent representation of Firmicutes, Bacteroidota, and Proteobacteria. (D) Rank‐abundance curves (log scale) showing long‐tailed community structure across individual samples, grouped by arm. (E) OTU/species accumulation curve across the mechanistic subset as a function of the number of samples sequenced. (F) Ternary plot at the phylum level illustrating arm‐skewed compositional positioning; point size reflects mean abundance. Sample IDs SA∗ = Con, SB∗ = Rif, SC∗ = Rif + Pro.

Microbiome profiling was performed in a prespecified mechanistic subset (total *n* = 20: Con *n* = 7, Rif *n* = 7, Rif + Pro *n* = 6). In figures where sample IDs are used, SA corresponds to Con, SB to Rif, and SC to Rif + Pro (SA=Con, SB=Rif, SC=Rif + Pro). Across this subset, the rank‐abundance curves display the expected long‐tailed ecological structure with pronounced dominance of a small number of taxa and a substantial rare tail (Figure 1D). The species/OTU accumulation curve rises steadily with increasing sample number and does not show an abrupt early plateau (Figure 1E), indicating that (within the limits of the subset size) additional sampling would be expected to capture additional low‐abundance features; importantly, the observed group differences described below are therefore interpreted as comparative patterns within the captured community structure rather than as claims of exhaustive diversity capture.

At the compositional level, stacked abundance displays and the clustered heatmap show treatment‐associated patterning rather than random intermixing (Figure 1A,B). The clustered heatmap reveals group‐linked blocks of higher versus lower relative abundance across multiple phyla/major lineages, and the clustering structure indicates that some samples cocluster preferentially by arm (Figure 1B), consistent with intervention‐associated ecological states. The Circos visualization further emphasizes that between‐arm differences are not confined to a single lineage but reflect multiphylum redistribution, with prominent contributions from Firmicutes, Bacteroidota, and Proteobacteria across samples and arms (Figure 1C). The ternary phylum plot similarly shows that several lineages occupy arm‐skewed regions of compositional space rather than being uniformly centered (Figure 1F), reinforcing the presence of group‐associated compositional bias.

Alpha diversity shifts are not monotonic and suggest distinct ecological “states” rather than simple diversity restoration.

**FIGURE 2:**
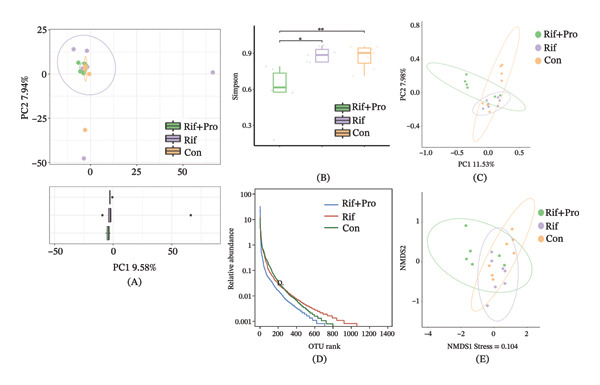
Diversity structure and ordination‐based separation of fecal microbiota across arms. (A) PCoA plot with arm‐colored points and ellipse overlays; the inset shows PC1 distribution across arms, highlighting outlier behavior. Axes report PC1 and PC2 variance explained. (B) Alpha diversity (Simpson index) compared across Con, Rif, and Rif + Pro; brackets and asterisks indicate significant contrasts as shown. (C) Alternative PCoA view with ellipses illustrating arm‐specific centroid and dispersion differences. (D) Group‐level rank‐abundance summaries comparing tail behavior across arms. (E) NMDS ordination with ellipses; stress is reported on the panel.

Simpson diversity differs across arms (Figure 2B), and the directionality is notable: the Rif + Pro group shows a markedly lower Simpson index compared with both Rif and Con, while Rif and Con exhibit higher and more similar values. This pattern argues against a simplistic “more diversity is better” narrative and instead suggests that combination therapy may drive a distinct ecological configuration characterized by stronger dominance or reduced evenness, despite the clinically favorable neurometabolic gradient. In other words, the data support a model in which functional or taxon‐specific restructuring, rather than global diversity gain, tracks the treatment gradient.

Beta diversity demonstrates arm separation with influential outliers and different within‐group dispersion.

Ordination plots show separation by arm with visible differences in within‐group dispersion. In PCoA space (Figure 2A), most points cluster tightly near the origin with an evident outlier along PC1 and additional outliers along PC2, indicating that a small number of samples exert strong leverage on the 2‐D representation. The PC1 distribution inset (Figure 2A, lower panel) confirms that the outlier behavior is not purely visual artifact but corresponds to genuinely shifted PC1 scores. Alternative PCoA representation with ellipses (Figure 2C) displays a distinct ellipse geometry and orientation across arms, suggesting that the three groups differ not only in the centroid location but also in the covariance structure (i.e., how variation is distributed within each arm). NMDS similarly shows arm‐structured separation with stress 0.104 (Figure 2E), supporting acceptable low‐dimensional representation of the dissimilarity structure and providing convergent evidence that group differences are robust to ordination method.

### 3.1. Feature Selection and Targeted Validation Identify Interpretable Taxa Consistent With the Treatment Gradient

**FIGURE 3:**
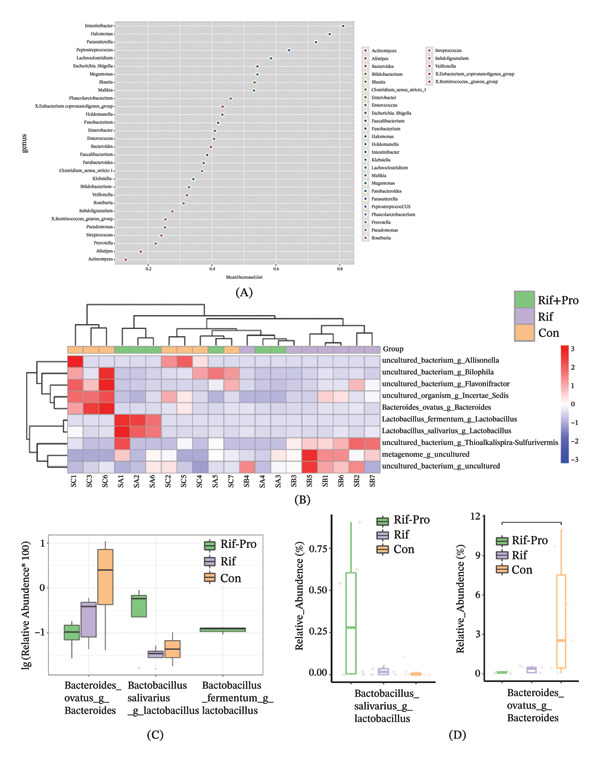
Machine‐learning feature ranking and validation of key taxa linked to arm separation. (A) Random forest variable importance plot (MeanDecreaseGini) identifying the genera contributing most strongly to arm discrimination. (B) Heatmap of selected discriminative taxa across samples with hierarchical clustering; top annotation indicates treatment arm. (C) Group comparison of representative taxa (including Bacteroides ovatus and Lactobacillus salivarius and fermentum) shown as boxplots of (log) relative abundance. (D) Focused boxplots for Lactobacillus salivarius and Bacteroides ovatus highlighting directionality consistent with the overall treatment gradient.

A random forest model trained at the genus level ranks discriminative features by MeanDecreaseGini, highlighting a set of taxa that contribute most strongly to arm separation (Figure 3A). The highest‐ranked features include genera such as Intestinibacter and Halomonas, followed by multiple taxa spanning Firmicutes, Proteobacteria, and Bacteroidota, indicating that the discriminatory signal is multilineage rather than restricted to a single clade. Heatmap clustering of selected features shows structured abundance blocks across samples that align with group annotation (Figure 3B), supporting that these ranked taxa are not isolated anomalies but participate in coordinated patterns.

Targeted comparisons in the same figure provide biologically interpretable directionality for key taxa. Bacteroides ovatus shows a group gradient, with the highest abundance in Con, intermediate abundance in Rif, and markedly reduced abundance in Rif + Pro (Figure 3C,D), consistent with suppression or ecological displacement under combination therapy. In contrast, Lactobacillus salivarius demonstrates the opposite pattern, with a clear increase in Rif + Pro relative to the other arms and broader dispersion within Rif + Pro (Figure 3C,D), consistent with treatment‐linked ecological reconfiguration. Lactobacillus fermentum appears concentrated in Rif + Pro (Figure 3C), consistent with treatment‐linked detection/enrichment; however, given 16S resolution limits, this is interpreted as an ecological signal rather than definitive proof of strain‐level engraftment.

Phylogenetic enrichment patterns further distinguish arms and support multilayered restructuring.

**FIGURE 4:**
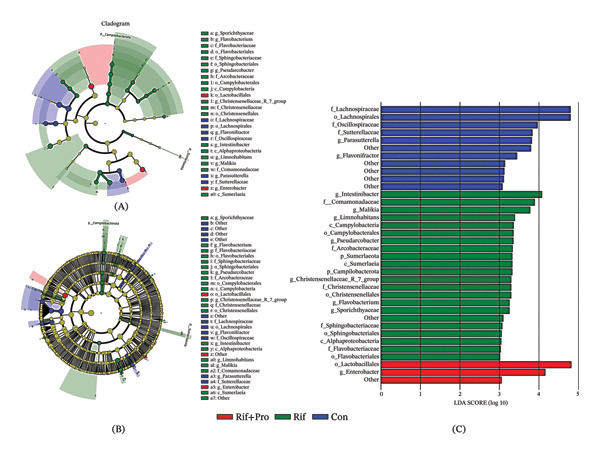
LEfSe‐identified discriminant taxa mapped to phylogeny and summarized by effect size. (A‐B) Cladogram visualizations of discriminant features showing phylogenetic clustering of enriched taxa by arm (Con, Rif, Rif + Pro). (C) LDA score (log10) bar plot showing the strongest enriched features for each arm, providing an effect‐size ranking of discriminant taxa.

LEfSe cladograms show that discriminatory taxa map onto specific phylogenetic branches rather than occurring as random isolated points (Figure 4A,B). LDA score ranking provides an effect‐size view of the features most enriched in each arm (Figure 4C). Con is characterized by enrichment of multiple members of the Lachnospiraceae/Lachnospirales and Oscillospirales lineages, consistent with a Firmicutes‐dominant signature. Rif is characterized by enrichment of Intestinibacter and several Proteobacteria‐associated or aquatic/soil‐associated lineages (e.g., Comamonadaceae/Malikia/Limnohabitans and Campylobacteria‐related entries shown in the bar list), suggesting that rifaximin exposure is associated with a distinct feature set rather than a simple attenuation of Con‐like taxa. Rif + Pro shows strong enrichment of Lactobacillales and *Enterobacter* (Figure 4C), aligning with the targeted taxon results and providing phylogenetic consistency for the observed Rif + Pro signature.

### 3.2. Indicator ASV Analysis Identifies Arm‐Specific Signatures Beyond Differential Abundance

**FIGURE 5:**
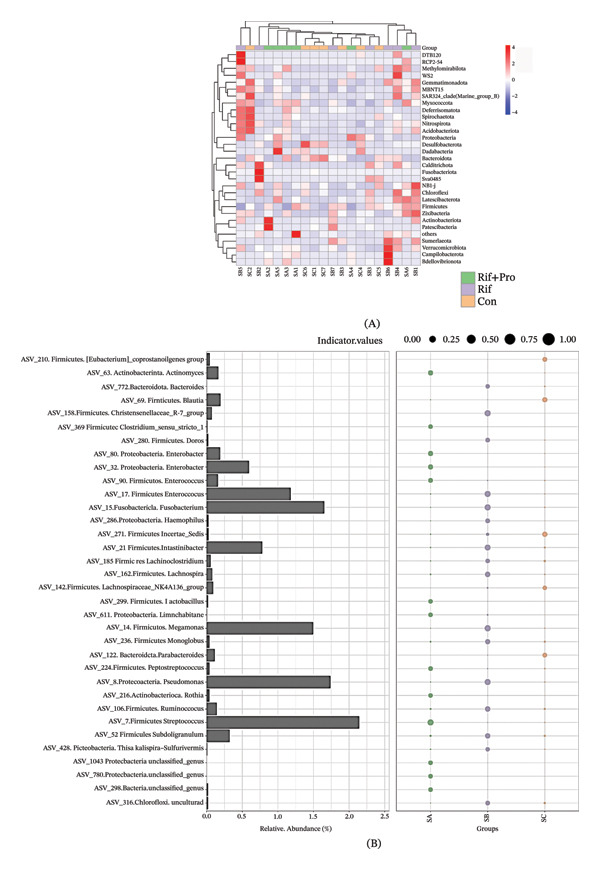
Arm‐specific signatures highlighted by lineage heatmap and indicator ASV analysis. (A) Heatmap displaying standardized abundance patterns across samples with arm annotation strip and hierarchical clustering. (B) Indicator analysis: the left panel displays relative abundance (%) for listed ASVs; the right panel shows indicator values by arm (bubble size indicates indicator strength), identifying ASVs with higher specificity to individual arms.

Heatmap visualization at the phylum/major lineage level (Figure 5A) demonstrates structured patterns across samples that correspond to the arm annotation strip, further supporting that the group signal is not driven by a single dominant lineage but by coordinated shifts across multiple lineages. Indicator value analysis (Figure 5B) identifies ASVs with stronger specificity to individual arms, shown by bubble size and distribution across the three group columns. Several indicator ASVs belong to Proteobacteria‐associated taxa (e.g., *Enterobacter*/*Haemophilus* entries visible in the ASV list), while others derive from Firmicutes‐associated taxa (e.g., *Enterococcus* and multiple Lachnospiraceae‐related entries), indicating that group specificity is distributed across both potentially proinflammatory and fermentative lineages. This indicator framework complements LEfSe by emphasizing arm‐specificity (signal uniqueness) rather than only differential abundance.

Taxonomic consistency across hierarchical levels is demonstrated in Supporting Figures S1–S3.

To ensure that the observed group patterns are not an artifact of a single taxonomic resolution, compositional displays were expanded across class (Supporting Figure S1), order (Supporting Figure S2), and family (Supporting Figure S3) levels. Across these hierarchical views, arm‐structured compositional patterning persists: grouped stacked bars show that each arm occupies a distinct compositional profile rather than appearing as permutation of the same structure, and the heatmap at the class level in S1A reveals block patterns aligned with arm labels, supporting that the group signal is detectable across multiple taxonomic granularities. These supporting analyses strengthen the inference that the intervention effects reflect broad ecological restructuring rather than a single taxonomic labeling choice.

Serum SCFA profiling reveals a selective propionate signal with broader pattern shifts.

**FIGURE 6:**
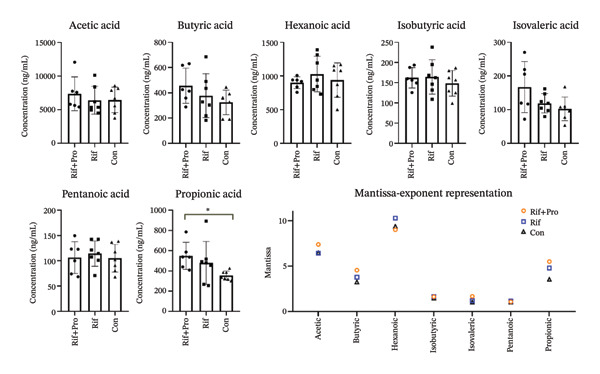
Serum short‐chain fatty acid profiling in the mechanistic subset. Bar plots show serum concentrations (ng/mL) of measured SCFAs (acetic, propionic, butyric, isobutyric, isovaleric, pentanoic, and hexanoic acids) with individual datapoints overlaid where available. Brackets and asterisks indicate displayed exploratory between‐group comparisons, notably for propionate. The Mantissa‐exponent representation summarizes multimetabolite patterns across arms.

Seven serum SCFAs were quantified in the mechanistic subset with individual datapoints displayed (Figure 6). Among these, propionic acid was higher in Rif + Pro than in Con, while Rif generally showed intermediate values. Butyric acid and isovaleric acid showed upward, nonsignificant differences in Rif + Pro relative to Con; remaining assayed SCFAs did not differ at the alpha = 0.05 level. These findings are interpreted as a selective serum metabolic‐output signal rather than global SCFA restoration, consistent with the exploratory mechanistic design.

Mucosal immune surveillance (SIgA) increases with therapy intensity and shows a positive exploratory association with propionate.

**FIGURE 7:**
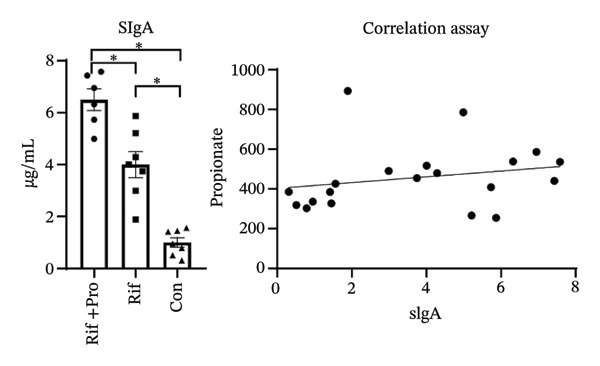
Fecal SIgA elevation by therapy and exploratory association with serum propionate. Left: fecal SIgA concentrations (μg/mL) across Rif + Pro, Rif, and Con with significance brackets as displayed. Right: exploratory Spearman association between fecal SIgA and serum propionate across the mechanistic subset with fitted trend line (*ρ* = 0.41; approximate two‐sided *p* = 0.072). This association is interpreted as supportive biological plausibility rather than definitive inferential evidence.

Fecal secretory IgA (SIgA) demonstrates a pronounced arm gradient (Figure 7). Rif + Pro shows the highest SIgA levels, Rif is intermediate, and Con is lowest, with multiple pairwise contrasts indicated by the bracketed asterisks. This pattern aligns directionally with the neurometabolic gradient (Table 3) and with the propionate increase (Figure 6). Correlation analysis showed a positive exploratory association between fecal SIgA and serum propionate across the mechanistic subset (Spearman *ρ* = 0.41; approximate two‐sided *p* = 0.072; Figure 7, right). This association is interpreted as supportive biological plausibility rather than definitive inferential evidence.

## 4. Discussion

This prospective three‐arm study examined whether a multistrain probiotic added to rifaximin, on the background of guideline‐concordant routine management for cirrhosis, is associated with incremental neurometabolic and mechanistic improvement in adults with HE. Across the cohort, the combination regimen tracked with a graded neurometabolic improvement relative to rifaximin alone, with stepwise reductions in post‐treatment serum ammonia (Con 177 ± 43.2; Rif 143 ± 37.5; Rif + Pro 117 ± 34.3 μmol/L) and lower exploratory composite HE indices (Con 10.0 ± 4.9; Rif 5.3 ± 4.7; Rif + Pro 3.7 ± 4.1). These readouts are not positioned as definitive clinical endpoints, but their monotonic gradient provides a coherent anchor against which the ecological, metabolic, and mucosal immune findings can be interpreted. The central contribution of the study is therefore not simply that the combination arm “changed the microbiome,” but that it generated a coordinated signal across multiple layers of the gut–liver–brain axis, including community‐level separation (PERMANOVA *p* = 0.006), discriminant microbial features, selective increases in serum propionate, and robust elevations in fecal SIgA with a positive exploratory association with propionate. Taken together, the data support a framework in which rifaximin‐associated ecological pressure and probiotic‐associated niche modulation jointly shift the intestinal ecosystem toward a distinct functional configuration that is biologically plausible for HE, even while causal inference remains out of scope for the present design [1, 3, 12–21].

Feature‐level analyses further strengthen the interpretation that the observed group differences are structured and biologically interpretable rather than being artifacts of a single plot type. Discriminant feature discovery identified arm‐associated taxa that clustered within phylogenetic context, and indicator approaches provided an additional specificity lens by highlighting ASVs preferentially associated with particular interventions. These analyses reduce the risk that interpretation depends on any one analytic lens. Within the set of interpretable taxa highlighted in the manuscript, Bacteroides ovatus decreased most strongly in the combination arm, while Lactobacillus salivarius increased, and Lactobacillus fermentum was detected in the combination arm. These directions are consistent with a model in which combined ecological pressure reshapes available niches and competitive dynamics, but they should not be overinterpreted as proof of durable colonization by administered strains. 16S rRNA sequencing does not confirm strain identity, does not resolve engraftment, and can misattribute closely related taxa depending on reference databases and classifier behavior [23]. Accordingly, the correct inference is ecological: the combination regimen was associated with a community state in which certain Lactobacillus‐associated features are more prominent and B. ovatus–associated features are less prominent, consistent with an altered niche structure under combined intervention pressures [23, 24]. Future work using strain‐resolved methods, including shotgun metagenomics with assembly‐based approaches, would be required to test whether administered strains persist and whether their persistence mediates host‐relevant outputs [23, 24].

The serum metabolite results are important because they translate ecological change into host‐relevant biochemical output. SCFAs are widely regarded as functional integrators of the microbiota–host communication. They support colonocyte energetics, reinforce tight junctions, calibrate mucosal and systemic immune tone, influence hepatic metabolism, and can signal along neuroimmune pathways relevant to brain function and neuroinflammation [12–15]. In HE, depletion of fermentative capacity and reduced SCFA production are plausible contributors to barrier failure, microbial translocation, inflammatory tone, and downstream cerebral vulnerability [1, 16]. In our mechanistic subset, propionic acid increased significantly in the combination arm relative to control, while butyrate and isovalerate showed upward trends without reaching statistical significance. This selective pattern matters. It suggests that combination therapy may not globally normalize all fermentative outputs but can shift specific metabolic pathways measurably, even under the constraints of modest sample size and interindividual variability. It is also consistent with a model in which rifaximin reduces harmful microbial metabolic activity and dysbiotic nitrogen flux, while probiotics and niche remodeling may facilitate reconstitution of cross‐feeding networks that preferentially restore certain fermentation products. This is a clinically relevant point: in the gut–liver–brain axis, a selective metabolic shift may be sufficient to alter barrier and immune signaling even if the community does not revert to a presumed “healthy” taxonomic baseline [12–16, 25].

The mucosal immune signal provides a complementary layer that is underrepresented in human HE studies. SIgA is a core mediator of immune exclusion at mucosal surfaces, coating microbes and antigens, limiting epithelial adhesion and penetration, and shaping microbial community structure while attenuating inflammatory exposure [17–20]. Cirrhosis‐associated immune dysfunction is characterized by both impaired surveillance and pathological activation, and impaired mucosal immune containment can plausibly exacerbate systemic inflammation and neurotoxic burden in HE [17–20]. In this context, the magnitude of SIgA elevation with active therapy is notable: approximately threefold in the rifaximin arm and nearly sixfold in the combination arm relative to controls, with the largest effect in the arm with the strongest neurometabolic improvement. The positive exploratory association between SIgA and serum propionate (Spearman *ρ* = 0.41; approximate two‐sided *p* = 0.072) supports biological plausibility but does not establish a statistically definitive association or causality. Prior human studies have shown probiotic‐associated augmentation of mucosal IgA in specific contexts, although effects are strain‐specific and dependent on host factors [16, 21]. Our findings extend this concept into a cirrhosis‐associated HE cohort and suggest that SIgA can be a sensitive mechanistic readout for future intervention trials in this population [17–21].

A recurring clinical concern in HE research is how to interpret ammonia and composite indices. We agree with the prevailing view that serum ammonia and composite scoring systems are imperfect proxies for clinical outcomes and should not substitute for event‐driven endpoints such as HE‐related rehospitalization, transplant, or death. In this manuscript, ammonia is treated analytically as a continuous biomarker and the composite HE index is explicitly exploratory. Nonetheless, the consistent stepwise pattern across both measures provides a pragmatic neurometabolic gradient that can be compared against mechanistic layers. The conceptual value lies in coherence: the arm with the lowest ammonia and lowest composite index also showed a distinct ecological state, a propionate increase, and the largest SIgA elevation. This multilayer alignment is the key observation. It supports the hypothesis that microbiota‐targeted combination therapy can produce coordinated shifts across the gut–liver–brain axis that track with neurometabolic improvement, even when the trial is not powered or designed to prove mediation [12–20, 22, 23, 25–27]. The appropriate next step is therefore not to overinterpret ammonia changes but to use the observed coherence to design a definitive, event‐driven trial with prespecified mechanistic endpoints and a rigorous analytic plan that tests whether mechanistic shifts statistically contribute to clinical benefit.

Safety and feasibility are inseparable from interpretation in cirrhosis, particularly when probiotics are used. The inclusion of *Enterococcus* faecium in the probiotic formulation raises a legitimate concern given the infection risk and immune dysfunction in advanced liver disease. The study prespecified surveillance, including early symptom monitoring, monthly laboratory testing, and blood cultures if febrile, and no bacteremia signal was observed. While this cannot exclude rare events, it provides a feasibility signal and a design template: future trials should embed systematic infection monitoring and maintain conservative eligibility criteria, and they should prespecify adverse‐event adjudication rules to avoid post hoc ambiguity. This is not merely procedural. For microbiota‐targeted strategies to be clinically credible in HE, safety governance must be part of the mechanistic narrative because barrier dysfunction and translocation risk are central to the disease biology itself [14, 16–20, 22].

Several limitations define interpretation and motivate the next‐generation study design. The total cohort size is modest, and the mechanistic subset is small, which limits precision and increases vulnerability to the influence of outlier samples in ordination space. This makes it essential to treat the mechanistic findings as exploratory, hypothesis‐generating signals and to prioritize replication under larger, prospective sampling. The microbiome profiling relies on 16S rRNA sequencing, which constrains strain‐level attribution and functional inference. Shotgun metagenomics or metatranscriptomics, combined with targeted pathway quantification, would sharpen biological interpretation, particularly for nitrogen metabolism, urease activity, bile‐acid transformations, and endotoxin‐associated pathways that are directly relevant to HE pathogenesis [23]. Similarly, metabolomics could be expanded beyond SCFAs to include amino‐nitrogen intermediates and bile‐acid derivatives, which are mechanistically linked to hepatic metabolism, microbial ecology, and neuroinflammatory tone [12–16]. Detailed lactulose dose‐by‐visit exposure was not modeled as a time‐varying covariate, and residual confounding from background lactulose titration cannot be fully excluded. Finally, because this study is not designed to formally test mediation, future trials should prespecify longitudinal sampling and causal‐inference frameworks that can evaluate whether changes in propionate, SIgA, or specific microbial feature sets statistically mediate event‐based outcomes.

Despite these limitations, the present study provides a coherent translational signal that informs trial design. The data support an event‐driven study in stabilized outpatients with recent overt HE history, using background lactulose in all arms and randomization to rifaximin with or without probiotic. The primary endpoint should be time to HE‐related rehospitalization or death over 6–12 months, adjudicated by blinded hepatologists, with psychometrics and quality of life as secondary endpoints. A mechanistic core should be prespecified and powered as secondary outcomes, including shotgun metagenomics for strain‐resolved ecology, targeted metabolomics including SCFAs and bile acids, markers of translocation and inflammatory tone, and mucosal immune measures such as SIgA. Safety monitoring should be tailored to cirrhosis, particularly if probiotic formulations include organisms with theoretical bacteremia risk. Power calculations should be anchored in expected recurrent‐HE event rates under combination therapy and incorporate prespecified multiplicity control for mechanistic endpoints [22–27].

In conclusion, adding a multistrain probiotic to rifaximin, on top of the guideline‐concordant routine management, was associated with a graded neurometabolic improvement compared with rifaximin alone, alongside structured ecological reconfiguration, selective elevation of serum propionate, and robust fecal SIgA increases with a positive exploratory SIgA–propionate association. These coordinated microbial, metabolic, and mucosal immune signals provide a biologically plausible, hypothesis‐generating rationale for microbiota‐targeted combination therapy in cirrhosis‐associated HE and justify confirmation in fully powered, event‐driven trials with deeper functional and strain‐resolved profiling.

## Author Contributions

Yiping Wang conceived and designed the analysis; Yiping Wang, Luna Lee, Hui Zhu, Shujun Wang, Danni Hu, and Mengdan Xu collected the data; Yiping Wang, Luna Lee, Hui Zhu, Shujun Wang, Danni Hu, and Mengdan Xu performed the analysis; Yiping Wang supervised the project; Yiping Wang and Luna Lee wrote the manuscript.

## Funding

This study was supported by grants from One clinical specialty construction program (grant numbers available upon request).

## Disclosure

All the authors gave written consent before publication of the work.

## Ethics Statement

Ethical approval was obtained from the Ethics Review Committee.

## Consent

All patients in this manuscript have given written informed consent for participation in the study and the use of their deidentified, anonymized, aggregated data, and their case details for publication.

## Conflicts of Interest

The authors declare no conflicts of interest.

## Supporting Information

Additional supporting information can be found online in the Supporting Information section.

## Supporting information


**Supporting Information 1** Supporting Figure S1. Class‐level ecological structure across arms. (A) Class‐level heatmap with hierarchical clustering and arm annotation demonstrates arm‐structured abundance blocks across samples. (B) Grouped stacked bar plots summarize class‐level composition within each arm, showing distinct compositional profiles rather than uniform mixing.

**Supporting Information 2** Supporting Figure S2. Order‐level composition across individual samples and grouped by arm. (A) Order‐level stacked bars across all samples (SA∗ = Con, SB∗ = Rif, SC∗ = Rif + Pro). (B) Grouped stacked bars summarize order‐level composition within each arm, supporting persistence of arm‐structured composition at this taxonomic resolution.

**Supporting Information 3** Supporting Figure S3. Family‐level composition across individual samples and grouped by arm. (A) Family‐level stacked bars across all samples (SA∗ = Con, SB∗ = Rif, SC∗ = Rif + Pro). (B) Grouped stacked bars summarize family‐level composition within each arm, demonstrating that arm‐linked composition patterns remain evident at the family level.

## Data Availability

The authors agree to make data and materials supporting the results or analyses presented in their paper available upon reasonable request. It is up to the author to determine whether a request is reasonable.
