# Supplementary figures and images for: Rifaximin Plus Probiotics Reshape Gut Microbiota, Serum Propionate, and Mucosal Immunity in Cirrhosis‐Related Hepatic Encephalopathy Microbiota‐Immune Remodeling in HE
Source: Can J Gastroenterol Hepatol. 2026 Jul 13;2026:2389961. doi: 10.1155/cjgh/2389961 (PMC13364995; doi:10.1155/cjgh/2389961)

## Slide 1
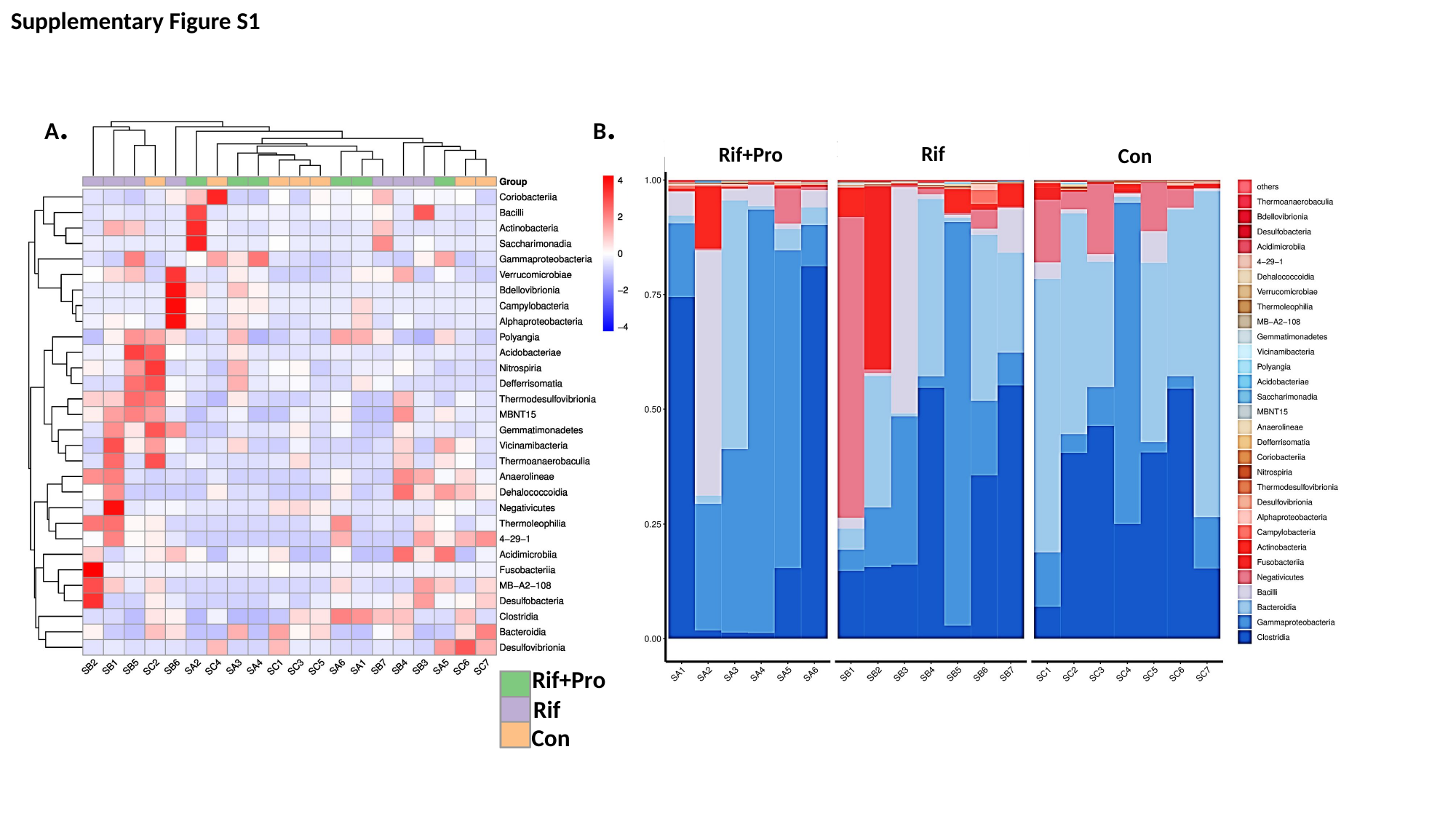

Supplementary Figure S1
A.
B.
Rif
Rif+Pro
Con
Rif+Pro
Rif
Con

Supplement: Supplementary file 1 — Supporting Information 1 Supporting Figure S1. Class‐level ecological structure across arms. (A) Class‐level heatmap with hierarchical clustering and arm annotation demonstrates arm‐structured abundance blocks across samples. (B) Grouped stacked bar plots summarize class‐level composition within each arm, showing distinct compositional profiles rather than uniform mixing. [file CJGH-2026-2389961-s001.ppt]

## Slide 1
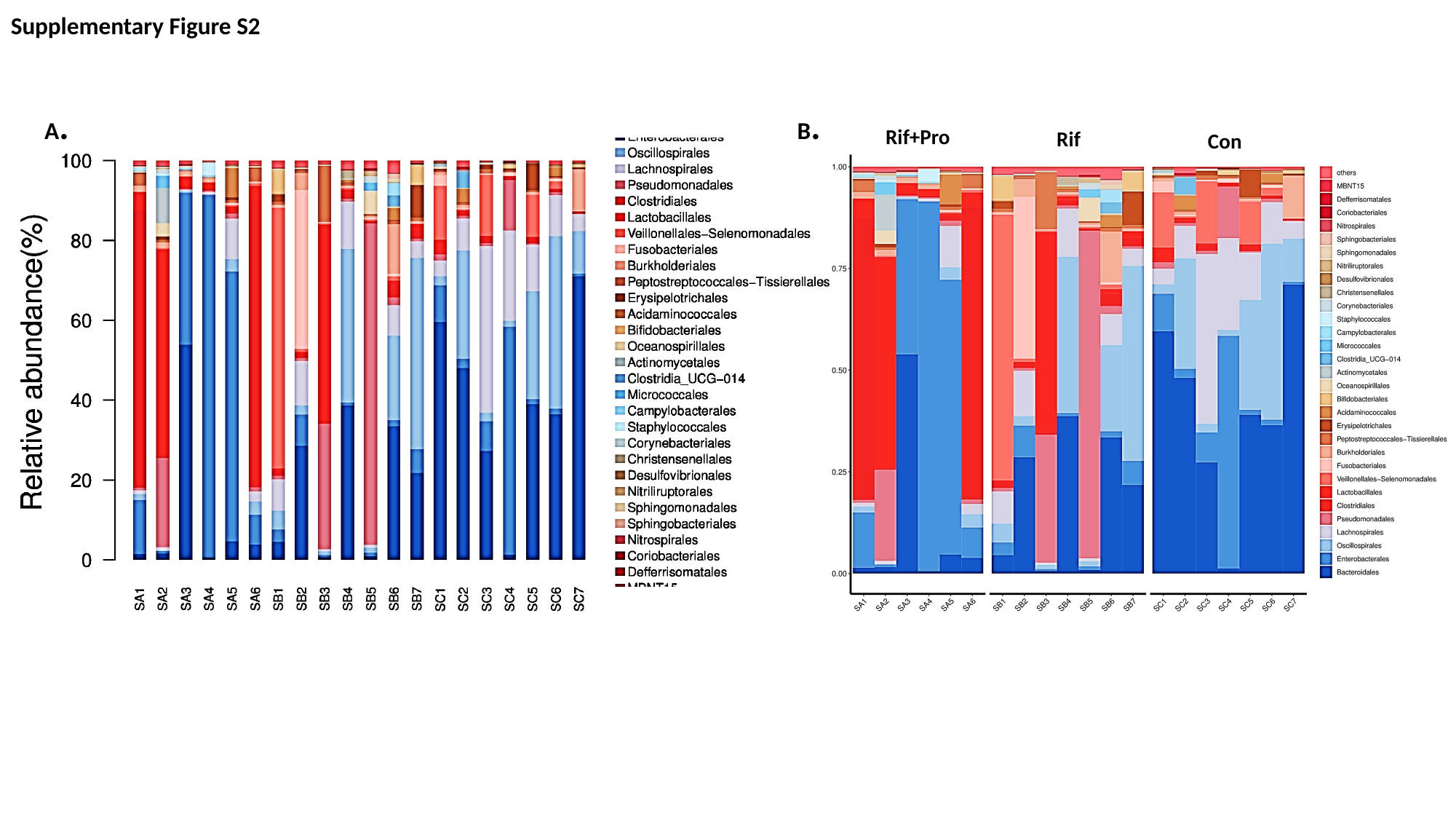

Supplementary Figure S2
A.
B.
Rif+Pro
Rif
Con

Supplement: Supplementary file 2 — Supporting Information 2 Supporting Figure S2. Order‐level composition across individual samples and grouped by arm. (A) Order‐level stacked bars across all samples (SA∗ = Con, SB∗ = Rif, SC∗ = Rif + Pro). (B) Grouped stacked bars summarize order‐level composition within each arm, supporting persistence of arm‐structured composition at this taxonomic resolution. [file CJGH-2026-2389961-s002.ppt]

## Slide 1
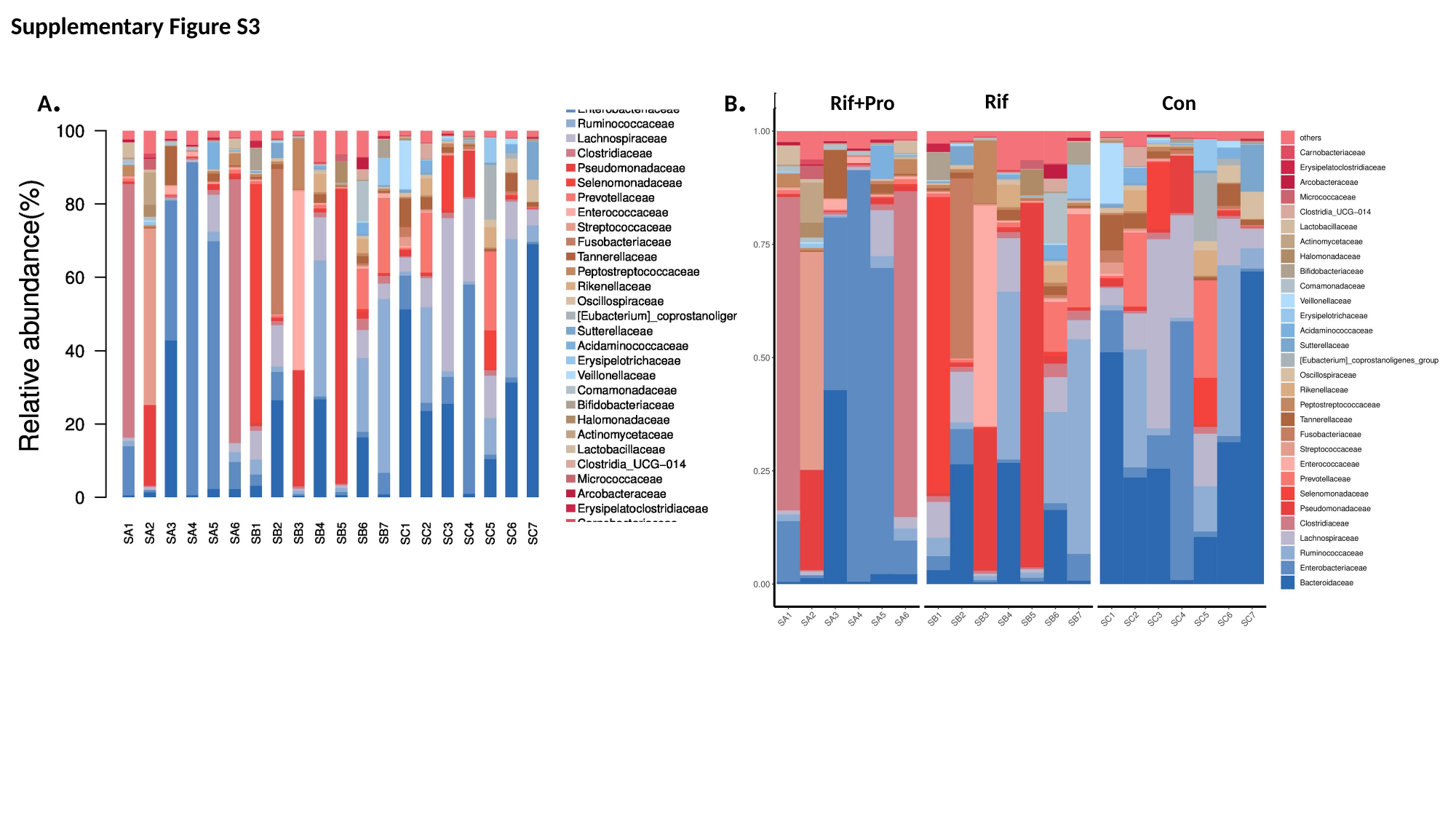

Supplementary Figure S3
A.
B.
Rif
Rif+Pro
Con

Supplement: Supplementary file 3 — Supporting Information 3 Supporting Figure S3. Family‐level composition across individual samples and grouped by arm. (A) Family‐level stacked bars across all samples (SA∗ = Con, SB∗ = Rif, SC∗ = Rif + Pro). (B) Grouped stacked bars summarize family‐level composition within each arm, demonstrating that arm‐linked composition patterns remain evident at the family level. [file CJGH-2026-2389961-s003.ppt]
